# Response accuracy of ChatGPT 3.5 Copilot and Gemini in interpreting biochemical laboratory data a pilot study

**DOI:** 10.1038/s41598-024-58964-1

**Published:** 2024-04-08

**Authors:** Ahmed Naseer Kaftan, Majid Kadhum Hussain, Farah Hasson Naser

**Affiliations:** 1 Biochemistry Department, Faculty of Medicine, Kufa University, Najaf, Iraq; 2 Najaf Health Directorate, Ministry of Health, Baghdad, Iraq

**Keywords:** Artificial intelligence models, ChatGPT-3.5, Copilot, Gemini, Biochemical parameters, Interpretation, Diagnosis, Information technology

## Abstract

With the release of ChatGPT at the end of 2022, a new era of thinking and technology use has begun. Artificial intelligence models (AIs) like Gemini (Bard), Copilot (Bing), and ChatGPT-3.5 have the potential to impact every aspect of our lives, including laboratory data interpretation. To assess the accuracy of ChatGPT-3.5, Copilot, and Gemini responses in evaluating biochemical data. Ten simulated patients' biochemical laboratory data, including serum urea, creatinine, glucose, cholesterol, triglycerides, low-density lipoprotein (LDL-c), and high-density lipoprotein (HDL-c), in addition to HbA1c, were interpreted by three AIs: Copilot, Gemini, and ChatGPT-3.5, followed by evaluation with three raters. The study was carried out using two approaches. The first encompassed all biochemical data. The second contained only kidney function data. The first approach indicated Copilot to have the highest level of accuracy, followed by Gemini and ChatGPT-3.5. Friedman and Dunn's post-hoc test revealed that Copilot had the highest mean rank; the pairwise comparisons revealed significant differences for Copilot vs. ChatGPT-3.5 (*P* = 0.002) and Gemini (*P* = 0.008). The second approach exhibited Copilot to have the highest accuracy of performance. The Friedman test with Dunn's post-hoc analysis showed Copilot to have the highest mean rank. The Wilcoxon Signed-Rank Test demonstrated an indistinguishable response (*P* = 0.5) of Copilot when all laboratory data were applied vs. the application of only kidney function data. Copilot is more accurate in interpreting biochemical data than Gemini and ChatGPT-3.5. Its consistent responses across different data subsets highlight its reliability in this context.

## Introduction

Medical experts are responsible for making sense of test results. Since this position requires diverse skills and deep medical understanding, it would be impossible to tailor it to each patient. There has been a rise in the demand for artificial intelligence models to assist those individuals because this could result in a missed diagnosis or incorrect interpretation^1^. Artificial intelligence (AI)-powered natural language processing (NLP) tools like ChatGPT-3.5, Google Gemini, and Microsoft Copilot. Numerous capabilities of various NLP tools have been examined. However, there has been a lack of investigation into their efficacy and precision in analyzing laboratory results^2^.

AI models have attracted much attention in the healthcare industry, and preliminary studies have shown encouraging outcomes. ChatGPT-3.5, developed by OpenAI, demonstrated remarkable pieces of evidence as a tool to aid clinical care with results that were comparable to those of the US Medical Licensing Examination (USMLE)^3–5^. AI programs are incredibly good at mimicking human speech patterns and producing natural-sounding responses to conversational text. Many people are curious about using ChatGPT and similar models in healthcare services. Both the AIs and the clinical experts who evaluated them had substantial training in clinical data. Specifically, AIs will most likely be used by patients and doctors to help them understand how to use clinical laboratory services and how to interpret laboratory data^6^. The current investigation aimed to evaluate how well three artificial intelligence models, ChatGPT-3.5, Copilot, and Gemini, interpret biochemical data from laboratory assessments. Thus, AI is relatively young; it has only been around for a year and is expanding phenomenally, making this study unique and groundbreaking.

## Methods

Ten simulated patients' biochemical laboratory data, including serum urea, creatinine, glucose, cholesterol, triglycerides, LDL-c, and HDL-c, in addition to HbA1c, have been selected for simulated patients in the laboratory of the Department of Biochemistry, Faculty of Medicine, University of Kufa in August 2023. No ethical clearance is necessary since these data are based on hypothetical patients. Table 1 displays the laboratory-selected parameters.

**Table 1:** Laboratory data of ten simulated patients, including serum urea, creatinine, glucose, cholesterol, triglycerides, LDL-c and HDL-c, and HbA1c.

	Urea (mmol/l)	Creatinine (µmol/l)	Glucose (mmol/l)	Cholesterol (mmol/l)	Triglycerides (mmol/l)	LDL-c (mmol/l)	HDL-c (mmol/l)	HbA1c (mmol/mol)
1	19.6	150.314	5.25	3.8	1.2	2.02	1.1	39
2	15.4	106.104	5.1	3.6	0.9	1.98	1.02	32
3	38.5	353.68	11.5	5	2.1	3	0.78	68
4	70	707.36	21	6.2	2.7	4.2	0.64	121
5	11.55	61.894	4.45	2.8	0.88	1.6	1.3	29
6	28	53.052	4.95	3.4	1.1	1.8	1	30
7	13.3	79.578	9.5	3.8	1.6	1.9	0.9	53
8	14	221.05	6	4.4	1.7	2.2	0.88	48
9	13.3	88.42	5.05	4.1	3.1	3.2	0.82	38
10	4.2	353.68	3.5	4.6	1.56	3.4	0.76	64

The data of the ten simulated patients were introduced to three AI models, Google ChatGPT-3.5, Copilot and Gemini, in association with the following question: "Please interpret the following laboratory tests, recommend further examinations, and provide a differential diagnosis. The study was carried out using two approaches. The first encompassed all the data presented in Appendices 1 and 2. The second approach involved the analysis of only data on kidney function, i.e., urea and creatinine levels, mentioned in Appendix 2. For a comprehensive and impartial evaluation, the answers of the AI programs were reviewed by a panel of three licensed independent medical professionals. For every outcome, there was a general agreement. The raters used a score ranging from 1 to 5 to indicate the accuracy of each generated answer. The detailed method of scoring follows:

- **Score 5:** Extremely accurate; the AI's response is spot on and by all current medical knowledge and best practices.
- **Score 4:** Reliable; the AI's response is largely accurate, with only minor inconsistencies that do not affect its clinical dependability.
- **Score 3:** Roughly correct; a human doctor or nurse may need to clarify or confirm the AI's answer because it has a few things that could be corrected.
- **Score 2:** Absence of data analysis.
- **Score 1:** Wrong; the AI's response needs to be corrected.

The final step of the current investigation involved comparing the mean rank scores of the AI model responses to all laboratory data with those of only kidney function data.

### Statistical analysis

IBM SPSS Statistics for Windows, version 25, was used for statistical analysis (IBM Corp., Armonk, New York, United States, https://www.ibm.com/spss). The Shapiro–Wilk test was used to check if the rater evaluations followed a normal distribution. Scores were found to be non-normally distributed; therefore, they were presented as medians and quartiles. Friedman and Dunn's post hoc analyses were used to compare the means of the scores pairwise. The Wilcoxon signed-rank test was used to compare the responses of each model separately in a pairwise fashion. A statistically significant result was defined as a P-value less than 0.05.

## Results

### Analysis of AI model responses when all laboratory data of ten simulated patients was applied

The AI models' response length (words) is shown in Table 2. The three AI programs showed a variety of answers, ranging from 286 to 890 words. The accuracy scores that the raters assessed were evaluated. Table 3 consists of the median and quartile values of the accuracy scores of the three AI models. Copilot had the highest level of accuracy (median: 5), according to the raters, followed by Gemini (median: 3), and then ChatGPT-3.5 (median: 2). The performance of the AI models was evaluated using Friedman and Dunn's post-hoc analyses. Table 4 shows that Copilot achieved the highest mean rank (2.95), indicating its overall superior performance compared to the other models. However, the pairwise comparisons revealed significant differences for Copilot vs. ChatGPT-3.5 (*P* = 0.002) and Gemini (*P* = 0.008).

**Table 2:** Response length generated by AI models used in data analysis.

AI model	Response length (words)
ChatGPT-3.5	286
Copilot	890
Gemini	290

**Table 3:** Median and quartiles of accuracy score of AI model responses assessed by the raters.

	N	Median	25th percentile	75th percentile
ChatGPT-3.5	10	2.0	2.0	3.0
Copilot	10	5.0	4.0	5.0
Gemini	10	3.0	1.0	4.0

**Table 4:** The mean rank of scores and pairwise comparison between the AI model's responses to laboratory data of ten simulated patients.

AI model	Mean rank	Pairwise comparison	Adjusted significance *P* value
ChatGPT-3.5	1.45	ChatGPT-3.5 vs Copilot	0.002
Copilot	2.95	Copilot vs Gemini	0.008
Gemini	1.60	Gemini vs ChatGPT-3.5	1.00

### Analysis of AI model responses when only kidney function data from ten simulated patients was applied

The accuracy of the responses of the three AI models to interpret only kidney function data, urea, and creatinine levels of the ten simulated patients was determined. Table 5 contains the median and quartile values of the accuracy scores of the three AI models. Again, the raters indicated that Copilot had the highest accuracy (median: 5), followed by Gemini (median: 4) and ChatGPT-3.5 (median: 4). The performance of the AI models was evaluated using Friedman and Dunn's post-hoc analyses to explore which response of the three AIs was the best. Table 6 shows that Copilot showed the highest mean rank (3.0), followed by Gemini (1.6) and ChatGPT-3.5 (1.4).

**Table 5:** The median and quartiles of accuracy scores of AI model responses to urea and creatinine results assessed by the raters.

	N	Median	25th percentile	75th percentile
ChatGPT-3.5	10	4	3.75	4.00
Copilot	10	5	4.75	5.00
Gemini	10	4	3.00	4.00

**Table 6:** The mean rank of scores and pairwise comparison between the AI model's responses to kidney function data of ten simulated patients.

AI model	Mean rank	Pairwise comparison	Adjusted significance *P* value
ChatGPT-3.5	1.4	Copilot vs ChatGPT-3.5	0.001
Copilot	3.0	Copilot vs Gemini	0.005
Gemini	1.6	Gemini vs ChatGPT-3.5	1.00

### Comparison of AI model responses for all laboratory data vs. those for only kidney function

The Wilcoxon Signed-Rank Test was used to compare the mean rank scores of the AI model responses to all laboratory data vs. only kidney function data (Table 7). Copilot demonstrated an indistinguishable response (*P* = 0.5) when all laboratory data were applied vs. the application of only kidney data. However, significant differences were evident in the responses of ChatGPT-3.5 (*P* = 0.02) and Gemini (*P* = 0.03) during similar analyses.

**Table 7:** The mean rank comparison of scores for AI responses to all tests and to renal tests only.

AI model	Mean rank	*P* value
Gemini	Negative rank: 0 Positive rank: 3	0.03
Copilot	Negative rank: 2 Positive rank: 2	0.5
ChatGPT-3.5	Negative rank:2 Positive rank: 4.86	0.02

## Discussion

To our knowledge, the current study is the first to compare the accuracy responses of three AI models, ChatGPT-3.5, Copilot, and Gemini, in interpreting biochemical data. Two approaches to accuracy determination were followed. The first included the assessment of the accuracy and response of all the biochemical parameters, consisting of kidney function data, urea, and creatinine, in the ten simulated patients. The second encompassed only the ten simulated patients' kidney function data, urea, and creatinine. Moreover, the accuracy of the responses obtained from the two approaches is compared. A panel of three experts reviewed the responses and provided feedback based on their evaluations. Copilot exhibited the highest accuracy rate compared to ChatGPT-3.5 and Gemini through the three rationales. These results suggest that the Copilot bot is a promising tool for analyzing laboratory biochemical data.

### Comparison of all biochemical data responses

The response length variation of the three AI models pointed out varied patterns. This variability highlighted the AI's own approach to generating answers. Some of them are more concise, and others are more detailed. Copilot demonstrated the highest length of word response. Several explanations could be addressed for this observation. Longer answers could signify a more detailed response to a question. Copilot's principal function is that of a search engine, and most of the stuff it returns is indexed from the internet. It seeks to offer pertinent data from multiple sources. Extended answers could come from providing a variety of ideas or combining several points of view^7^. Copilot, ChatGPT, and Gemini, all use different algorithms. The goals and features of Copilot are distinct from those of the other two models, resulting in distinct response content, style, and length. Expectations from users are essential. While some consumers value thorough explanations, others prefer succinct responses. Copilot may target people who value thoroughness with its longer answers^8^. Context-rich answers may rank higher on Copilot, particularly for intricate or multidimensional queries. However, accuracy is only sometimes improved by more extended responses. Quantity is not as important as quality. Thus, evaluating the responses' accuracy, applicability, and usefulness is essential^9^.

The assessment of the median and quartile values of the accuracy scores of all biochemical data (Approach 1) revealed Copilot to have the highest accuracy, followed by Gemini and then ChatGPT-3.5. It is essential to clarify that Copilot's primary purpose is to retrieve information from the internet, just like any other search engine. It is not intended for in-depth medical data analysis, although it can offer pertinent information on clinical and biochemical subjects^10^. Its capacity to compile data from numerous sources, such as reliable medical websites, research articles, and clinical guidelines, may account for its high ranking^11^. Gemini's moderate rating suggests that it performs reasonably well, while ChatGPT's performance is the lowest among others. Factors contributing to each AI model rating could include its training data, algorithmic approach, and validation against real-world clinical cases^12^.

Dunn's post-hoc analysis was used to evaluate the methodology and estimate the AI models' performance after the Friedman test. This statistical method assists in comparing several models and locating noteworthy variations in their functionality^13^. The Copilot's performance was the highest, as the mean rank was the highest. This suggests that Copilot fared better overall than the other models in interpreting clinical biochemical data. Pairwise comparisons revealed significant differences between Copilot and ChatGPT-3.5 and between Gemini and Copilot. This suggests that when it came to clinical biochemical data interpretation, Copilot performed more accurately than Gemini and ChatGPT-3.5^14^. The results, however, did not specifically identify the causes of this discrepancy.

### Comparison of kidney function data responses

The accuracy of the three AI models in interpreting only kidney function data (Approach 2) was estimated. This step is performed to verify if responses are affected when all data or a related part is applied. Again, Copilot demonstrated the highest accuracy among other AI models. However, Gemini and ChatGPT-3.5's performances seemed to improve. These results affirmed the superiority of Copilot in biochemical data interpretation. In addition, they explore response consistency when all or just a part of the data is introduced, i.e., whole biochemical data versus only kidney function data. Such findings remain immature and may need further verification and validation.

The mean rank of scores of the three AI models in interpreting kidney function data, urea and creatinine levels, were compared, followed by Dunn's post-hoc analysis. Copilot was found to have the highest mean rank, indicating better performance than Gemini and ChatGPT-3.5. The pairwise comparison with Gemini explores a significant difference in favor of Copilot. The lowest mean rank of ChatGPT3.5 suggests suboptimal performance. These findings highlight two conclusions. The first is Copilot’s superiority in interpreting kidney function data, urea, and creatinine levels. The second is the consistency of the studied AI models in responding when whole data or apart from is introduced for interpretation.

### All laboratory data vs. kidney function data comparison of responses

The Wilcoxon Signed-Rank Test was used to identify variations in AI model responses when comparing two related datasets^15^. The first set includes all data responses, which includes a broader set of information. The second contains only kidney function data, which is a more specific subset of data. Copilot revealed an indistinguishable response when considering all laboratory data versus only kidney data. However, other AI models seemed to respond differently when all data or those of kidney function were analyzed. These findings suggested that Copilot's response remained relatively similar whether all laboratory data or only kidney data were used^16^. Thus, two conclusions could be obtained from the Wilcoxon signed rank test findings. The first is that Copilot is a better AI model for interpreting biochemical data than Gemini and ChatGPT-3.5. The second is that its consistent responses across different data subsets highlight its reliability in this context.

### Comparison with previous studies of AI analysis of laboratory data

One of our challenges in the current study was the need for similar previous investigations with a compatible design. However, this challenge is incredible, as it gives the study pioneering status as it is being conducted for the first time in the field of using AI in analyzing laboratory results. Noteworthy, the relevant studies available on the Internet were searched to link what was reached in this study with those reported previously. Stevenson et al. evaluated the responses of ChatGPT-3.5 and Gemini to thyroid function tests in fifteen fictional cases. They have suggested that these tools could not consistently generate correct interpretation and could not be used as an alternative to the decisions of biochemists^17^. Bunch et al. surveyed previous studies of AI applications in clinical chemistry. They have pointed out that AI is a promising field across all testing phases, including pre-analytic, analytic, and post-analytic phases^18^. Mitra et al. reviewed research papers investigating the use of AI in clinical chemistry. They demonstrated that AI is promising for advancing laboratory medicine^19^. Azarkhish et al. developed a logarithm for predicting iron deficiency anemia from complete blood count results^20^. Luo et al. applied a logarithm to explore ferritin levels from AI information obtained from extracted clinical laboratory data^21^. Lee et al. highlighted a protocol for estimating LDL-c using a dataset of deep neural networks consisting of total cholesterol, HDL-c, and triglyceride levels. They concluded that their model is more accurate than other methods like the Friedewald equation and other novel methods^22^.

In conclusion, Copilot is more accurate at interpreting biochemical data than Gemini and ChatGPT 3.5. Its consistent responses across different data subsets highlight its reliability in this context. Further studies are essential to validate the findings.

### Limitations of the study

Several limitations were identified in the study. The first is the limited study sample; the study relied on ten simulating hypothetical patients and may not fully represent the diverse and complex clinical cases in the real world. An increased number of hypothesized and more diverse cases could enhance research results. The second is the rater’s subjectivity; evaluators' ratings are subjective in nature, and raters' decisions can affect the reliability of the results. Increasing inter-rater agreement and implementing additional validation methods increases the likelihood of achieving optimality and enhances study reliability. The third is that the interpretation of results for patients in a purely statistical matter may limit the reliability of the results. We have to emphasize that our findings represent a starting point and that clinical judgment, patient context, and individualized care remain essential components in the real-world application of AI systems. The fourth is the lack of external validation; the study relied on hypothetical data that may not entirely resemble patient data in real-world clinical scenarios. Improving the reliability of the results and enhancing the study requires external validation using actual patient data. The fourth is the lack of temporal stability; the study does not consider the performance stability of AI models over time. The models' accuracy may fluctuate as medical practices evolve and new data becomes available.

### Declaration of generative AI and AI-assisted technologies in the writing process

The writers edited and proofread the document using AI models to make this work more readable. The authors assumed complete responsibility for the publication's content and revised and edited it as necessary.

### Supplementary Information

Supplementary Information 1.

Supplementary Information 2.

## Data Availability

The datasets used and analyzed during the current study are available from the corresponding author upon reasonable request.
