# Supplementary material for: Response accuracy of ChatGPT 3.5 Copilot and Gemini in interpreting biochemical laboratory data a pilot study
Source: Sci Rep. 2024 Apr 8;14:8233. doi: 10.1038/s41598-024-58964-1 (PMC11002004; doi:10.1038/s41598-024-58964-1)
Supplement: Supplementary file 1 — Supplementary Information 1. [file 41598_2024_58964_MOESM1_ESM.docx]

**Appendix 1**

**The responses for data interpretation by AI models are presented as follows:**

**Responses of ChatGPT-3.5**

“Interpreting these laboratory blood tests and providing a differential diagnosis and recommendations for further tests can help identify potential health issues. Let's analyze each set of results:

**Patient 1:**

Differential Diagnosis: These results indicate mildly elevated urea and creatinine levels, suggesting possible kidney dysfunction. The HbA1c level is in the prediabetic range, and the lipid profile is borderline high.

Recommendations: Further tests may include:

Kidney function tests (e.g., GFR) to assess kidney health.

Regular monitoring of blood sugar levels for diabetes diagnosis.

**Patient 2:**

Differential Diagnosis: These results are generally within the normal range, indicating good kidney function and blood sugar control.

Recommendations: Continue with routine health checks.

**Patient 3:**

Differential Diagnosis: These results suggest significant kidney impairment, poorly controlled diabetes, and an unfavourable lipid profile.

Recommendations: Immediate consultation with a healthcare provider for kidney evaluation and diabetes management. Assess cardiovascular risk.

**Patient 4:**

Differential Diagnosis: These results indicate severe kidney impairment, extremely high blood sugar levels (uncontrolled diabetes), and severe dyslipidemia.

Recommendations: Immediate medical attention and consultation with specialists for kidney failure and diabetes management. Assess cardiovascular risk.

**Patient 5:**

Differential Diagnosis: These results suggest normal kidney function, well-controlled blood sugar levels, and a favourable lipid profile.

Recommendations: Continue with routine health checks and a healthy lifestyle.

**Patients 6 to 10** generally have varying degrees of kidney function, blood sugar control, and lipid profiles. Based on the patient's medical history and clinical presentation, specific recommendations for further tests and diagnoses should be made. It's crucial to remember that laboratory results are just one part of the diagnostic process, and a comprehensive evaluation by a healthcare provider is necessary to determine the underlying causes and develop a personalized treatment plan for each patient.”

**Responses of Google Gemini:**

**“Patient 1**

This patient has average laboratory blood test results.

**Patient 2**

This patient has average laboratory blood test results.

**Patient 3**

This patient has elevated urea and creatinine levels, HbA1c and FBS levels, and LDL, cholesterol, and triglyceride levels. This suggests that the patient may have kidney disease, prediabetes or diabetes, and heart disease risk factors.

**Patient 4**

This patient has elevated urea and creatinine levels, HbA1c and FBS levels, and LDL, cholesterol, and triglyceride levels. These suggest severe kidney disease, diabetes, and heart disease risk factors. The patient may also be dehydrated.

**Patient 5**

This patient has normal laboratory blood test results.

**Patient 6**

This patient has normal laboratory blood test results.

**Patient 7**

This patient has elevated HbA1c and FBS levels as well as elevated LDL and cholesterol levels. This suggests that the patient may have prediabetes or diabetes and heart disease risk factors.

**Patient 8**

This patient has elevated creatinine levels and elevated LDL and cholesterol levels. This suggests that the patient may have kidney disease and heart disease risk factors.

**Patient 9**

This patient has elevated HbA1c and fasting blood glucose levels and elevated LDL, cholesterol, and triglyceride levels. This suggests that the patient may have prediabetes or diabetes and heart disease risk factors.

**Patient 10**

This patient has elevated urea and creatinine levels, HbA1c and FBS levels, and LDL, cholesterol, and triglyceride levels. This suggests that the patient may have kidney disease, prediabetes or diabetes, and heart disease risk factors.

Please note that these are just general recommendations based on the limited information provided. Further testing and evaluation by a doctor or other healthcare provider is necessary to make a definitive diagnosis and develop a treatment plan.”

**Responses of Copilot AI:**

“Hello, this is Copilot. I will try to interpret the laboratory blood tests you provided, recommend further tests, and give a differential diagnosis. Please note that these are not definitive diagnoses, and you should consult your doctor for confirmation and treatment.

**Patient 1:** This person has elevated urea and creatinine levels, which indicate impaired kidney function. The ratio of urea to creatinine is 33, which suggests a pre-renal cause of acute kidney injury, such as dehydration, heart failure, or shock. The other parameters are within the normal range or slightly above the normal range. Further tests to evaluate kidney function include urine analysis, urine osmolality, fractional excretion of sodium, and renal ultrasound. Possible differential diagnoses include hypovolemia, sepsis, cardiac dysfunction, or renal artery stenosis.

**Patient 2:** This person has normal blood test results, except for a slightly elevated creatinine level, which may indicate a mild decrease in kidney function or increased muscle mass. No further tests are needed unless there are symptoms or risk factors for kidney disease. Possible differential diagnoses include benign creatinine elevation, early chronic kidney disease, or muscle injury.

**Patient 3**: This person has very high urea and creatinine levels, which indicate severe kidney failure. The ratio of urea to creatinine is 27.5, which suggests a prerenal or intrinsic cause of acute kidney injury, such as volume depletion, nephrotoxic drugs, glomerulonephritis, or acute tubular necrosis. The other parameters are also elevated and indicate poorly controlled diabetes mellitus and dyslipidemia. Further tests to evaluate kidney function include urine analysis, urine osmolality, fractional excretion of sodium, and renal ultrasound. Further tests to evaluate diabetes mellitus and dyslipidemia include fasting lipid profile, urine microalbumin, serum electrolytes, and haemoglobin A1c. Possible differential diagnoses include diabetic nephropathy, acute interstitial nephritis, rhabdomyolysis, or multiple myeloma.

**Patient 4:** This person has extremely high urea and creatinine levels, which indicate end-stage renal disease. The ratio of urea to creatinine is 25, which suggests an intrinsic cause of chronic kidney injury, such as diabetic nephropathy, chronic glomerulonephritis, polycystic kidney disease, or interstitial fibrosis. The other parameters are also very elevated and indicate uncontrolled diabetes mellitus and severe dyslipidemia. Further tests to evaluate kidney function include urine analysis, urine osmolality, fractional excretion of sodium, and renal ultrasound. Further tests to evaluate diabetes mellitus and dyslipidemia include fasting lipid profile, urine microalbumin, serum electrolytes, and haemoglobin A1c. Possible differential diagnoses include diabetic nephropathy, chronic interstitial nephritis, amyloidosis, or lupus nephritis.

**Patient 5:** This person has normal blood test results, except for a slightly low creatinine level, which may indicate a low muscle mass or malnutrition. No further tests are needed unless there are symptoms or risk factors for muscle wasting or nutritional deficiency. Possible differential diagnoses include sarcopenia, anorexia nervosa, cachexia, or hyperthyroidism.

**Patient 6:** This person has elevated urea level and low creatinine level, which indicates a high protein intake or catabolism and a low muscle mass or malnutrition. The ratio of urea to creatinine is 133.3, which suggests a high protein breakdown or intake from diet or gastrointestinal bleeding. The other parameters are within the normal range or slightly above the normal range. Further tests to evaluate protein metabolism include serum protein electrophoresis, urine protein electrophoresis, and faecal occult blood tests. Possible differential diagnoses include multiple myeloma, gastrointestinal malignancy, liver cirrhosis, or starvation.

**Patient 7:** This person has normal urea and creatinine levels, which indicate normal kidney function. The other parameters are elevated and indicate diabetes mellitus and dyslipidemia. Further tests to evaluate these conditions include a fasting lipid profile, urine microalbumin, serum electrolytes, and haemoglobin A1c. Possible differential diagnoses include type 2 diabetes mellitus, metabolic syndrome, or Cushing’s syndrome.

**Patient 8:** This person has an elevated creatinine level, which indicates impaired kidney function. The ratio of urea to creatinine is 16, which suggests an intrinsic cause of acute or chronic kidney injury, such as nephrotoxic drugs, glomerulonephritis, or acute tubular necrosis. The other parameters are also elevated and indicate diabetes mellitus and dyslipidemia. Further tests to evaluate kidney function include urine analysis, urine osmolality, fractional excretion of sodium, and renal ultrasound. Further tests to evaluate diabetes mellitus and dyslipidemia include fasting lipid profile, urine microalbumin, serum electrolytes, and haemoglobin A1c. Possible differential diagnoses include diabetic nephropathy, acute interstitial nephritis, rhabdomyolysis, or multiple myeloma.

**Patient 9:** This person has normal urea and creatinine levels, which indicate normal kidney function. The other parameters are elevated and indicate dyslipidemia and borderline diabetes mellitus. Further tests to evaluate the dyslipidemia include fasting lipid profile and apolipoprotein levels. Further tests to evaluate diabetes mellitus include an oral glucose tolerance test and haemoglobin A1c. Possible differential diagnoses include familial hypercholesterolemia, familial hypertriglyceridemia, or metabolic syndrome.

**Patient 10:** This person has a very low urea and creatinine levels, indicating a low protein intake or synthesis and a high muscle mass or breakdown. The ratio of urea to creatinine is 3, which suggests a low protein synthesis or intake from liver disease or malnutrition. The other parameters are also elevated and indicate diabetes mellitus and dyslipidemia. Further tests to evaluate the protein metabolism include serum protein electrophoresis, urine protein electrophoresis, and liver function tests. Further tests to evaluate diabetes mellitus and dyslipidemia include fasting lipid profile, urine microalbumin, serum electrolytes, and hemoglobin A1c. Possible differential diagnoses include liver failure, malabsorption syndrome, muscle dystrophy, or hemodialysis.”

**Appendix 2**

**Responses of ChatGPT-3.5:**

Interpreting the laboratory blood tests with urea and creatinine values given in different units (urea in mmol/L and creatinine in μmol/L) can be challenging, but I'll provide an analysis based on the provided data. The normal reference ranges for these values may vary depending on the laboratory and the units used, so it's essential to consider the lab's reference range for accurate interpretation.

**Patient 1:**

Interpretation:

Elevated urea and creatinine levels may suggest impaired kidney function.

Recommendations:

Further tests may include a GFR (Glomerular Filtration Rate) to assess kidney function.

Monitor for any signs or symptoms of kidney disease.

**Patient 2:**

Interpretation:

Urea and creatinine levels are within or close to the normal range.

Recommendations:

Continue with routine health checks.

**Patient 3:**

Interpretation:

Elevated urea and creatinine levels indicate significant kidney impairment.

Recommendations:

Urgent consultation with a healthcare provider for kidney function evaluation.

Further tests may include imaging studies of the kidneys to assess their structure and function.

**Patient 4:**

Interpretation:

Extremely high urea and creatinine levels suggest severe kidney dysfunction or kidney failure.

Recommendations:

Immediate medical attention and referral to a nephrologist (kidney specialist) for evaluation and potential dialysis.

**Patient 5:**

Interpretation:

Urea and creatinine levels are within or close to the normal range.

Recommendations:

Continue with routine health checks.

**Patient 6:**

Interpretation:

Urea and creatinine levels are within or close to the normal range.

Recommendations:

Continue with routine health checks.

**Patient 7:**

Interpretation:

Urea and creatinine levels are within or close to the normal range.

Recommendations:

Continue with routine health checks.

**Patient 8:**

Interpretation:

Creatinine is significantly elevated, indicating potential kidney dysfunction.

Recommendations:

Further evaluation is needed, including a GFR and additional kidney function tests.

Consultation with a nephrologist for assessment and management.

**Patient 9:**

Interpretation:

Urea and creatinine levels are within or close to the normal range.

Recommendations:

Continue with routine health checks.

**Patient 10:**

Interpretation:

Urea is relatively low, but creatinine is significantly elevated, indicating potential kidney dysfunction.

Recommendations:

Further evaluation, including a GFR and additional kidney function tests, is necessary.

Consultation with a healthcare provider or nephrologist for assessment and management.

These interpretations and recommendations are based on the available data, but specific clinical evaluation and consideration of the lab's reference ranges are crucial for accurate diagnosis and management. Kidney function tests and imaging may be required to determine the underlying causes of abnormal results and guide further treatment.

**Responses of Google Gemini:**

“Interpretation of laboratory blood tests

**Patient 1:**

Interpretation: Elevated urea and creatinine levels suggest impaired kidney function.

**Patient 2:**

Interpretation: Urea and creatinine levels are within or close to the normal range.

**Patient 3:**

Interpretation: Elevated urea and creatinine levels indicate significant kidney impairment.

**Patient 4:**

Interpretation: Extremely high urea and creatinine levels suggest severe kidney dysfunction or kidney failure.

**Patient 5:**

Interpretation: Urea and creatinine levels are within or close to the normal range.

**Patient 6:**

Interpretation: Urea and creatinine levels are within or close to the normal range.

Patient 7:

Interpretation: Urea and creatinine levels are within or close to the normal range.

**Patient 8:**

Interpretation: Creatinine is significantly elevated, indicating potential kidney dysfunction.

**Patient 9:**

Interpretation: Urea and creatinine levels are within or close to the normal range.

**Patient 10:**

Interpretation: Urea is relatively low, but creatinine is significantly elevated, indicating potential kidney dysfunction.

Differential diagnosis

The differential diagnosis for elevated urea and creatinine levels includes:

Acute kidney injury (AKI)

Chronic kidney disease (CKD)

Heart failure

Liver disease

Dehydration

Obstruction of the urinary tract

Certain medications

Recommendations for further testing

Further testing may be recommended based on the patient's clinical presentation and the results of their initial laboratory tests. Some common tests that may be ordered include:

Glomerular filtration rate (GFR) test to assess kidney function

Electrolytes and urinalysis

Imaging studies of the kidneys, such as an ultrasound or CT scan

Management

The management of elevated urea and creatinine levels will depend on the underlying cause. In some cases, patients may require hospitalization and dialysis. In other cases, patients may be able to manage their condition with lifestyle changes and medication.

It is important to note that this is just an overview of the interpretation and management of elevated urea and creatinine levels. A qualified healthcare professional should make specific recommendations on a case-by-case basis.

**Responses of Copilot:**

The following is my interpretation of the laboratory blood tests based on the normal ranges of urea and creatinine in blood and urine. I also recommend further tests and give a differential diagnosis for each case.

1. The urea level is slightly elevated, while the creatinine level is normal. This could indicate mild dehydration, a high-protein diet, or a minor kidney impairment. Further tests to check the urine-specific gravity, osmolality, and electrolytes may help to confirm the cause. Differential diagnoses may include prerenal azotemia, urinary tract infection, or liver disease.

2. The urea and creatinine levels are both normal, indicating normal kidney function and hydration status. No further tests or differential diagnoses are needed.

3. The urea and creatinine levels are both significantly elevated. This indicates a severe kidney impairment or failure. Further tests to check the glomerular filtration rate (GFR), urine output, and blood pressure may help assess the damage's extent. Differential diagnoses may include acute tubular necrosis, glomerulonephritis, nephrotic syndrome, or chronic kidney disease.

4. The urea and creatinine levels are both extremely elevated. This indicates a critical kidney failure that requires urgent medical attention. Further tests to check the serum potassium, bicarbonate, calcium, and phosphate levels may help to monitor the risk of complications such as hyperkalemia, metabolic acidosis, or renal osteodystrophy. Differential diagnosis may include end-stage renal disease, rhabdomyolysis, or obstructive uropathy.

5. The urea level is normal, while the creatinine level is low. This could indicate low muscle mass, malnutrition, or pregnancy. Further tests to check the serum albumin, albumin, and total protein levels may help evaluate the nutritional status. Differential diagnoses may include cachexia, anorexia nervosa, or hyperthyroidism.

6. The urea level is elevated, while the creatinine level is low. This could indicate a liver dysfunction or damage that affects the conversion of ammonia to urea. Further tests to check the serum ammonia, bilirubin, alkaline phosphatase, and liver enzymes may help to diagnose the liver condition. Differential diagnoses may include cirrhosis, hepatitis, or hepatic encephalopathy.

7. The urea and creatinine levels are both normal, which indicates normal kidney function and hydration status. No further tests or differential diagnoses are needed.

8. The urea level is normal, while the creatinine level is elevated. This could indicate a high muscle mass, strenuous exercise, or muscle breakdown. Further tests to check the serum creatine kinase (CK), lactate dehydrogenase (LDH), and myoglobin levels may help to detect any muscle injury or inflammation. Differential diagnosis may include muscular dystrophy, myositis, or rhabdomyolysis.

9. The urea and creatinine levels are both normal. This indicates a normal kidney function and hydration status. No further tests or differential diagnosis are needed.

10. The urea level is low, while the creatinine level is elevated. This could indicate a severe liver dysfunction or damage that affects the production of urea from ammonia. Further tests to check the serum ammonia, bilirubin, alkaline phosphatase, and liver enzymes may help to diagnose the liver condition. Differential diagnosis may include acute liver failure, fulminant hepatitis, or hepatocellular carcinoma.”
